# The association between male infertility and sperm disomy: Evidence for variation in disomy levels among individuals and a correlation between particular semen parameters and disomy of specific chromosome pairs

**DOI:** 10.1186/1477-7827-2-82

**Published:** 2004-12-14

**Authors:** Helen G Tempest, Sheryl T Homa, Maria Dalakiouridou, Dimitra Christopikou, David Wright, Xiao P Zhai, Darren K Griffin

**Affiliations:** 1 Cell and Chromosome Biology Group, Department of Biological Sciences, Brunel University, Uxbridge, UK; 2 112 Harley Street, London, UK; 3 Medicines Control Agency, Market Towers, 1 Nine Elms Lane, London, UK; 4 Department of Biosciences, University of Kent, Canterbury, CT2 7NZ, UK

## Abstract

**Background:**

The association between infertility and sperm disomy is well documented. Results vary but most report that men with severely compromised semen parameters have a significantly elevated proportion of disomic sperm. The relationship between individual semen parameters and segregation of specific chromosome pairs is however less well reported as is the variation of disomy levels in individual men.

**Methods:**

In order to address these questions the technique of fluorescent in-situ hybridisation (FISH) was utilised to determine the disomy levels of chromosomes X, Y and 21 in 43 sperm samples from 19 infertile males. The results generated from this study were analysed using logistic regression.

**Results:**

In this study we compared levels of sperm concentration, motility and morphology with levels of sperm disomy for chromosome 21 and the sex chromosomes. Our results suggest that there is considerable variation in disomy levels for certain men. They also suggest that oligozoospermic males have significantly elevated levels of sex chromosome disomy but not disomy 21; they suggest that severe asthenozoospermic males have significantly elevated levels of disomy 21 but not sex chromosome disomy. Surprisingly, severe teratozoopsermic males appeared to have significantly lower levels of sperm disomy for both the sex chromosomes and chromosome 21.

**Conclusion:**

We suggest that the association between sex chromosome disomy and oligozoospermia may be due to reduced recombination in the XY pairing region and discuss the relevance of our findings for the correlations between sperm disomy and sperm motility and morphology.

## Background

The relationship between male infertility and elevated proportions of sperm with extra or missing chromosomes in any given ejaculate is now extensively documented. There have been over 30 studies that have investigated this effect [e.g. [1-7]], and the majority have suggested a highly significant relationship between decreased semen quality parameters and increased sperm disomy. At least three studies however [3,8,9] have suggested that there is only a moderate increase in disomy associated with male infertility and a further three have found no significant relationship [2,10,11]. The reasons for these apparent discrepancies between groups are not clear although they may reflect laboratory-specific differences in stringency of scoring criteria, collection of semen samples after different periods of abstinence and/or criteria for patient selection differing from study to study. An alternative explanation however is that, among individuals and individual patient cohorts, some men have elevated levels of sperm disomy associated with infertility whereas others do not. If this is the case, there are a number of possible explanations; perhaps environmental influences could play a role. Indeed, a number of synthetic chemicals have been shown to be able to mimic endogenous hormones and affect the normal pattern of reproductive development [12]. In humans, levels of sperm disomy can be increased by environmental factors such as alcohol abuse and heavy smoking [13,14]. Intrinsic factors such as age and DNA polymorphisms have also been implicated. Indeed age and its effect on sperm disomy is well established [15,16]; Abruzzo et al. [17] found no effect of Y chromosome alphoid array size on Y chromosome non-disjunction, however Hobbs et al. [18] recently identified a genetic polymorphism involved in folate metabolism as a significant risk factor for trisomy 21.

A number of authors [4,11,19-21] refer to "severe oligoasthenoteratozoospermia (OAT)." Pang et al. [4] defined OAT as a sperm concentration of less than 15 million per ml, motility of less than 41% and normal morphology of less than 4.4%. This phenotype has been associated with increases in sperm disomy levels of around tenfold compared to normal controls [4]. Other papers however are less descriptive about the semen parameters in their patient cohort, and few studies set out to establish any relationship between individual semen parameters and the frequency of disomy of specific chromosomes. Exceptions to this include two studies that have examined patients with teratozoospermia alone [7,22]. Further studies, demonstrated a negative correlation between sperm disomy for sperm concentration [7,23,24]. Correlations were also found between disomy and progressive motility [24,25], disomy and teratozoospermia [7,25]. Viville et al. [22] analysed four individual patients presenting with four different types of total teratozoospermia. In that study, no significant difference was reported for three patients however one patient with macrocephalic spermatozoa had an aneuploidy rate of around 90%, demonstrating a significant correlation with morphology for patients with macrocephalic spermatozoa.

In most of the above studies either semen parameters and or aneuploidies for individual chromosome pairs were grouped together and thus not considered individually. Moreover, cases where males have given multiple samples are rare and thus there are few occasions where the individual specific parameters have been compared on a sample-by-sample basis. Establishing chromosome-specific and parameter-specific correlations between male infertility and percentage of aneuploid sperm in an ejaculate is a preliminary step towards understanding the mechanisms of the association between male infertility and chromosome segregation. In this study, our results provide evidence for a variation in rates of disomy for individual men and a correlation between specific semen parameters and individual chromosome disomies.

## Methods

### Patient cohort and experimental design

A series of males undergoing infertility treatment with a range of andrological phenotypes were assessed for conventional semen parameters and for sperm disomy. All patients were attending IVF clinics in the central London area. Semen samples were taken, with patients' informed consent, from 19 different men on 43 occasions from infertility clinics in central London. None had known constitutional karyotypic abnormalities or Y chromosome deletions. We received 1 sample each from 12 men, 2 samples from one man, 3 samples from 2 men, 4 samples from one man, 6 samples from 2 men and 7 samples from one man. In some men one, two or three of the semen parameters measured (concentration, motility and morphology) were within the normal range; these were hence placed in a control group. In other cases (test group) individual parameters were in the abnormal range (see subsequent section for andrological criteria). Given that some samples were taken from individual patients on several occasions, sometimes males appeared in the control group for some samples and in the test group for others. We restricted our molecular cytogenetic studies to chromosome 21 and the sex chromosomes for three reasons. First, according to previous studies [26-28] these are the most prone to non-disjunction in sperm and hence the most likely to give significant results. Second, the sheer number of sperm that needed to be scored per individual to establish statistically significant results precluded the study of large numbers of chromosome pairs. Finally these pairs are the most clinically significant as they lead to common mutant phenotypes among liveborns. That is, unlike most trisomies that abort in the first trimester, trisomies of the sex chromosomes and chromosome 21 frequently go to term and can lead to Klinefelter Syndrome and Down Syndrome respectively.

### Semen analysis

Men were required to abstain from ejaculating for between 2 and 5 days prior to providing a sample for the study. Samples were produced on site into sterile 60 ml containers and kept at room temperature for up to 60 minutes to allow for liquefaction. Semen parameters were then analysed according to guidelines defined by WHO [29]. Patients were assessed for sperm quality using WHO guidelines and Kruger strict criteria for assessment of morphology. Concentration, percent motility, forward progression and the percentage of normal morphology were noted. The same operator performed all analyses. Sperm morphology was assessed on unstained samples, using phase contrast microscopy at a magnification of × 640. Evaluation of normal forms was based on Kruger strict criteria as described by Menkveld et al [30].

Individual samples were then placed in three occasions into a "test" or "control" group. On the first occasion, the control group had a sperm concentration of ≥ 20 million/ml and the test group <20 million/ml. On the second occasion, the control group had forward motility of ≥ 20% and the test group <20%. On the third occasion the control group had a normal morphology of ≥ 4% and the test group <4%. The cut-off points for considering individual samples as being in the test or control groups were based on WHO guidelines for oligozoospermia, severe asthenozoospermia, and severe teratozoospermia and were comparable to those used in other studies for sperm disomy [e.g. [4]]. In each case the sample was assessed by fluorescent in-situ hybridization (FISH) for the proportion of disomic sperm.

### FISH analysis

FISH analysis was performed according to Griffin et al [15]. Briefly samples were prepared as follows: samples were washed in a buffer solution (10 mM Tris HCl, 10 mM NaCl, pH 8.0), smeared onto clean microscope slides, and dehydrated in an alcohol series. Slides were then air dried, and sperm heads were swelled by successive incubations in 0.1 M DTT (30 minutes) and 0.1 M LIS (1 hour). Slides were then dehydrated in an alcohol series and air dried ready for subsequent FISH studies. Three colour FISH was carried out for chromosomes 21, X and Y in each patient using directly labelled commercially available probes (Vysis Inc., Downers Grove, Il, USA). Spectrum Orange LSI 21 DNA probe, Spectrum Green CEP Y (satellite III) DNA probe, and a combination of the CEP X centromeric alpha-satellite probes one labelled in Spectrum Orange and one in Spectrum Green were used to give a yellow colour. The protocol followed was identical to that of Griffin et al. [15] with the exception that the colour combinations (above) used.

Approximately 5,000 sperm were scored per patient by two or more independent observers. The proportion of aneuploid sperm per sample for each chromosome was noted.

### Statistical Analysis

The hypotheses of interest were whether the rate of disomy was significantly different for the test and control groups in terms of sperm concentration, morphology or motility. To this end, in the first case, all oligospermic patients designated "O" (i.e. O, OT, OA and OAT in table 1) were in the test group and the remainder were in the control group. In the second case, the test group were severe asthenozoospermic patients designated "A" (A, OA, AT and OAT – control group were the remainder) and, in the third case, the test group were severe teratozoospermic patients designated "T" (T, OT, AT and OAT – control group were the remainder). Thus six patients (5, 9, 10, 12, 14 and 17) appeared in more than one group on at least one occasion. Data on the rate of disomy were generated for chromosome 21 and the sex chromosomes. To test these hypotheses six logistic regression models were fitted in the statistical software package SAS (Version 8.2). For each model an Odds Ratio, a 95% confidence interval and a p-value were calculated. A confidence interval that does not contain 1 implies that there is evidence that the disomy rates were significantly different for that comparison and hence has a corresponding p-value < 0.05.

**Table 1:** Incidence of sperm disomy for the sex chromosomes and chromosome 21 and the semen analysis in 43 men.

**Patient number**	**% disomy sex chromosomes**	**% chromosome 21 disomy**	**Total cells scored**	**count (million/ml)**	**Motility (%)**	**% abnormal forms**	**Semen analysis**
1	0.50%	0.43%	5097	6.9	26	97	OT
2	1.02%	0.10%	5000	6.6	48	98	OT
3	0.29%	0.29%	4864	128	63	86	N
4	0.35%	0.20%	4790	13.5	17	98	OAT
5a	0.17%	0.24%	5400	61	24	95	N
5b	0.22%	0.10%	5092	37.5	7	95	A
5c	0.12%	0.10%	5000	22	45	99	T
5d	0.06%	0.14%	5000	63	13	94	A
6a	0.14%	0.08%	5000	48	44	98	T
6b	0.12%	0.04%	5000	40	50	99	T
7	0.18%	0.20%	5000	4.8	<10	95	OA
8	0.74%	0.00%	544	<0.01	<10	100	OAT
9a	0.73%	0.25%	5066	17	18	96	OAT
9b	0.45%	0.24%	5056	12	11	97	OAT
9c	0.30%	0.14%	5084	21	30	96	T
9d	0.10%	0.22%	5000	80	8	98	AT
9e	0.18%	0.12%	5063	13.3	26	97	OT
9f	0.24%	0.12%	5004	7.9	42	98	OT
10a	0.24%	0.38%	5000	25	48	99	T
10b	0.02%	0.04%	5000	8	38	97	OT
10c	0.16%	0.12%	5000	22	41	98	T
11	0.17%	0.09%	5275	41	46	93	N
12a	0.96%	1.46%	4050	69	10	91	A
12b	0.44%	0.11%	5449	10.3	26	87	O
12c	0.30%	0.12%	5048	9.5	11	97	OAT
12d	0.24%	0.14%	5018	63	17	97	AT
12e	0.18%	0.08%	5031	32	41	96	T
12f	0.12%	0.06%	5061	57	37	96	T
12g	0.14%	0.18%	5000	24	58	87	N
13	0.06%	0.02%	5000	96	60	92	N
14a	0.65%	0.20%	3521	12	36	97	OT
14b	0.24%	0.08%	5009	7.5	53	99	OT
14c	0.14%	0.10%	5001	37	27	97	T
14d	0.08%	0.14%	5037	16	45	98	OT
14e	0.20%	0.04%	5000	30	40	100	N
14f	0.27%	0.37%	4108	16	38	94	O
15	2.00%	0.59%	5293	14	57	96	OT
16	0.31%	0.21%	5120	47	49	89	N
17a	0.24%	0.24%	5000	18	53	92	O
17b	0.08%	0.04%	5000	49	44	96	T
17c	0.08%	0.04%	5000	44	56	92	N
18	0.10%	0.26%	5000	123	10	96	AT
19	0.30%	0.18%	5107	52	46	89	N

Patient number: Letters after patient numbers indicate consecutive samples from the same patient e.g. 10b is the second sample from patient 10

Semen Analysis: O = oligozoospermia (sperm concerntration <20 million/ml); A = severe asthenozoospermia (formward motility <40%); T = severe teratozoospermia (normal motility <4% by strict Kruger criteria); N = normozoospermia (>20 million/ml concentration; >40% formward motlity; >4% normal morphology).

## Results

**Figure 1:**
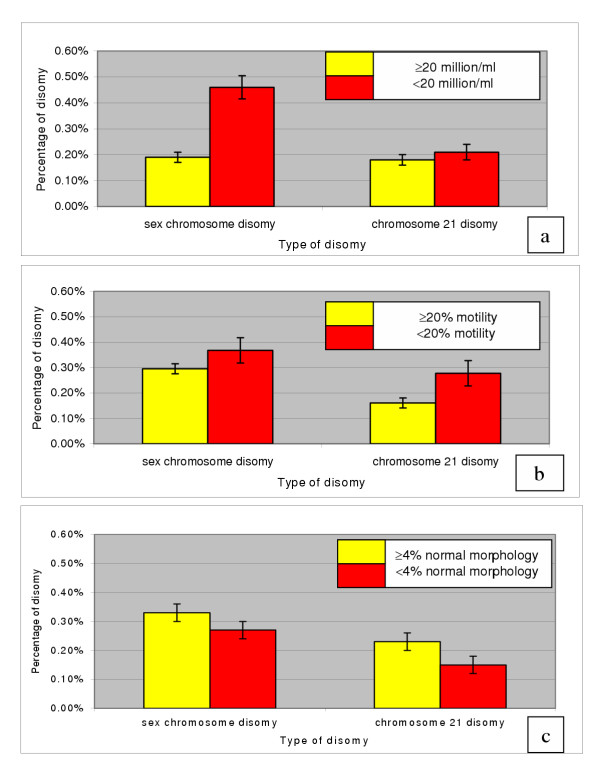
**a) **Mean sperm disomy frequencies for the sex chromosomes and chromosome 21 in two groups with sperm concentration of ≥ 20 million/ml and < 20 million/ml. **b) **Mean sperm disomy frequencies for the sex chromosomes and chromosome 21 in two groups with percentage of motility of ≥ 20% motility and < 20% sperm motility. **c) **Mean sperm disomy frequencies for the sex chromosomes and chromosome 21 in two groups with ≥ 4% normal morphology and < 4% normal morphology. Error bars represent SEM.

**Figure 2:**
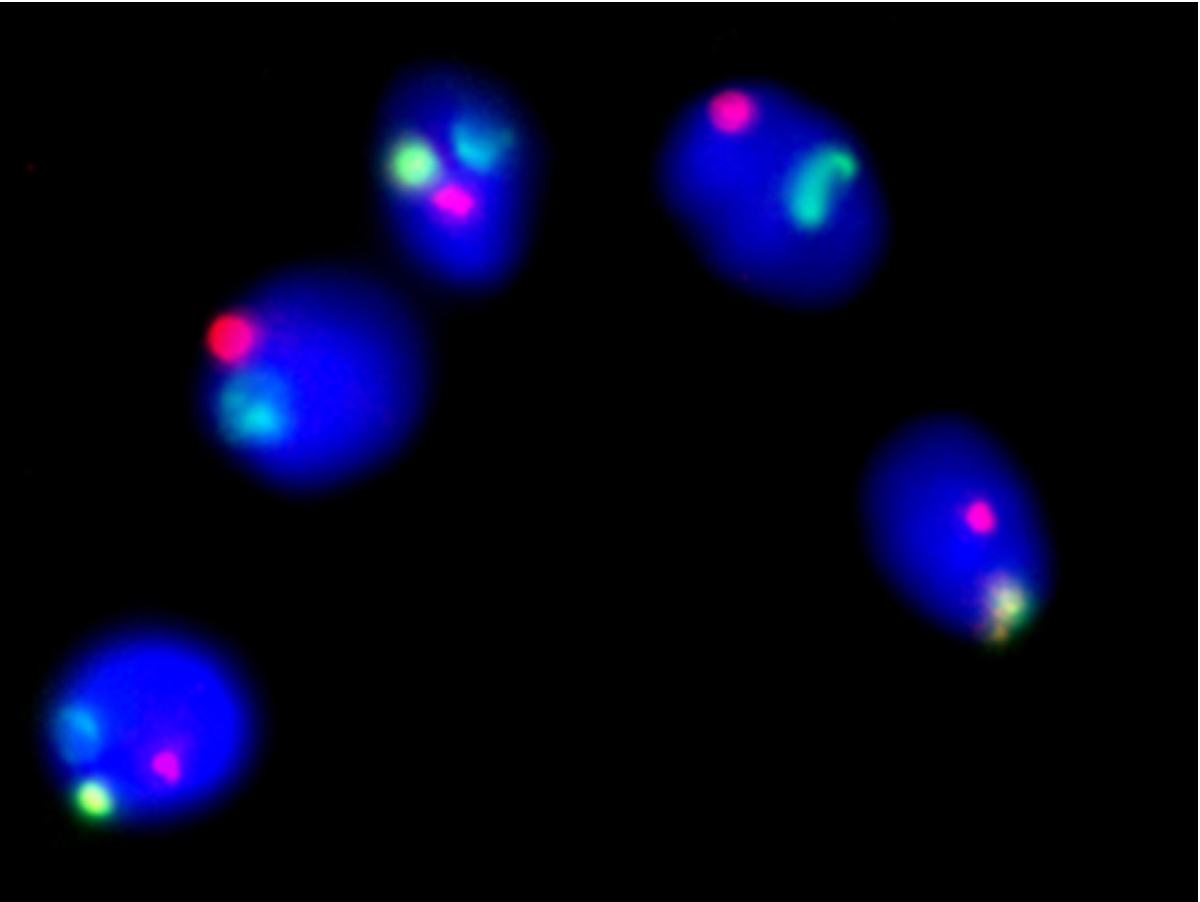
Image of five sperm with chromosome X labelled in yellow, Y in green and 21 in red. The sperm at the bottom left and top centre-left are XY disomic.

A total of 209,188 spermatozoa were scored (approximately 5,000 per sample). The total rate of disomy for the sex chromosomes and chromosome 21 was found to be 0.3% (633/209,188) and 0.19% (398/209,188) respectively. Disomy levels ranged from 0.02% – 2.00% for the sex chromosomes and 0.00% – 1.46% for chromosome 21. For the patients who gave multiple samples, individual disomy rates were surprisingly varied: For instance patient 9 (who gave 6 samples) had sex chromosome disomy frequencies ranging from 0.1% and 0.73%. The results of each individual sample are presented in Table 1 where, in each case, the semen parameters as well as the sperm disomy rates for each individual chromosome are given. The results of the logistic regression analysis (table 2) clearly demonstrate that men with oligozoospermia (figure 1a), (sperm concentration < 20 million/ml) have significantly elevated levels of sex chromosome disomy (Odds Ratio 2.39, p < 0.0001) in their sperm compared to men with normal sperm count levels (sperm concentration ≥ 20 million/ml). As the lower limit of the 95% confidence interval for the Odds Ratio is 2.04 these data suggest that the rate of sperm disomy is likely to be at least twice as high in test patients compared to controls. Further analysis revealed that this increase was largely accounted for by an increase in XY disomy, which is usually associated with non-disjunction errors of meiosis I (data not shown). Conversely there was no evidence of a significant association between oligozoospermia and sperm disomy for chromosome 21. For the motility data (figure 1b) however, the opposite situation pertained. That is, there was no significant difference between sperm disomy levels for the sex chromosomes (XY, XX or XY disomy) whereas men with motility of < 20% (asthenozoospermia) had significantly elevated levels of chromosome 21 disomy compared to controls (Odds Ratio 1.75, p < 0.0001), (table 2). Finally (and surprisingly) men with severe teratozoospermia (figure 1c), (< 4% abnormal forms) had significantly reduced levels of sperm disomy for both pairs of chromosomes compared to controls. (Sex chromosome disomy, Odds Ratio 1.22, p = 0.013, Chromosome 21 disomy, Odds Ratio = 1.54, p=<0.0001) (table 2). Figure 2 shows examples of normal and XY disomic sperm.

**Table 2:** Logistic regression analysis of individual disomy rates compared to semen parameters. a. Sperm concentration, b. sperm motility, c. sperm morphology. **(C) **= control values, (**T**) = test values.

**a. Sperm concentration**				
Chromosome	Mean disomy	SD (4 dp)	Odds Ratio (95% Confidence Interval)	p-value

21	(**C**) 0.19% (**T**) 0.20%	0.0018 0.0028	1.14 (0.94, 1.40)	0.18

Sex	(**C**) 0.20% (**T**) 0.48%	0.0046 0.0015	2.39 (2.04, 2.81)	< 0.0001

**b. Motility**				

Chromosome	Mean disomy	SD	Odds Ratio (95% Confidence Interval)	p-value

21	(**C**) 0.16% (**T**) 0.28%	0.0013 0.0037	1.75 (1.43, 2.14)	< 0.0001

Sex	(**C**) 0.30% (**T**) 0.37%	0.0037 0.0029	1.12 (0.95, 1.34)	0.19

**c. Morphology**				

Chromosome	Mean disomy	SD	Odds Ratio (95% Confidence Interval)	p-value

21	(**C**) 0.24% (**T**) 0.16%	0.0039 0.0013	1.54 (1.26, 1.89)	< 0.0001

Sex	(**C**) 0.33% (**T**) 0.28%	0.0039 0.0024	1.22 (1.04, 1.43)	0.013

## Discussion

To the best of our knowledge, this is the first report that demonstrates a relationship between individual clinically defined semen parameters and segregation of specific chromosome pairs. The relationship between sex chromosome disomy and the failure of spermatocytes to complete spermatogenesis is manifested by significantly higher sex chromosome disomy levels in oligozoospermic men. These results are similar to those of Rives et al. [23], Vegetti et al. [24], Calogero et al. [7] who also reported a relationship between sex chromosome disomy and sperm concentration. Here however we also report the absence of such an effect for chromosome 21 and this leads us to propose the hypothesis that this effect may be restricted to the sex chromosome bivalent. This could be confirmed by further studies using probes for different autosomes and studies are ongoing in this regard. A common mechanism leading to both XY disomy and failure of spermatogenesis is aberrant pairing in the pseudoautosomal region. That is, Hassold et al. [31] demonstrated that men with paternally derived Klinefelter syndrome arose through a reduction or absence in pairing in the pseudoautosomal region. Furthermore, the term "obligatory" has often been used to describe the need for X and Y to pair and recombine in order for spermatogenesis to proceed properly [32]. In the light of our results therefore we propose that a common mechanism leading to oligozoospermia and increased levels of XY disomy involves a perturbation in the mechanisms of synapsis and/or recombination in the XY paring region. In order to test this hypothesis, in future studies, we will use single sperm PCR [33] using primers both within and outside the XY paring region. A significant difference between oligozoospermic males compared to controls would provide evidence to support our hypothesis.

Our results also suggest a significant association between asthenozoospermia (poor motility) and non-disjunction of chromosome 21. This is similar to reports by Vegetti et al. [24] but in this case, we found no such association with the sex chromosomes. One possible explanation is that over expressed genes on chromosome 21 significantly impair the formation of the sperm midpiece through which sperm motility is mediated. This seems unlikely however since chromosome 21 is a gene-poor chromosome and there are thought to be few genes expressed in the spermatocyte itself that impact on spermiogenesis. It is also possible that there are gene products (e.g. micro tubular or motor proteins) common to both normal chromosome segregation of chromosome 21 (or the acrocentric (non-Y) chromosomes, or the autosomes in general) and normal formation of the structures that mediate sperm motility. We could establish the extent to which this effect is widespread in other autosomes by similar experiments using probes for other chromosomes; again these studies are ongoing. A final possibility is that our results represent a statistical anomaly. While correlations for individual males who have given multiple (four or more) samples are relatively consistent for sperm concentration, they are less so for motility (see table 1). If disomy were related to motility by a genetic cause, then would expect a consistency in chromosomal aneuploidies from individual patients who gave multiple samples. In patient 5 however, his highest motility sample of 45% also had the lowest proportion of autosomal disomy (0.1%) and the highest (0,24%) in a "normal" motility sample. There was also considerable evidence of varying disomy levels when motility remained relatively constant for instance patient 10, had normal motility in all samples, the disomy frequency ranged from 0.12 – 0.38%. Clearly therefore further studies are necessary before a stronger relationship between autosomal sperm disomy and asthenozoospermia can be established.

The apparent inverse association between sperm morphology and chromosome segregation was surprising and it is, again, possible that this is a statistical anomaly. Indeed although a number of studies have found no significant correlation between morphology and disomy [24,34-36], others suggest a positive correlation between disomy and abnormal morphology [7,25]. The high level of significance, the fact that the effect is clear in two separate chromosome pairs and the fact that different effects were seen for concentration and morphology would argue that this is a genuine phenomenon. Moreover this is one of the few studies that has used repeat samples from individual patients and, in some cases, the same individual appeared in different groups depending on his semen parameters at the time of donation. In other words disomy levels appear not to be consistent among individuals, rather they relate more to their semen parameters on any given day, perhaps as a result of extrinsic factors. Other studies have reported that teratozoospermic males have elevated levels of sperm disomy, Calogero et al. [7] found a correlation between increased sperm disomy levels and teratozoospermia as did Viville et al. [22] but only an association with macrocephalic spermatozoa. For the most part however these individuals were defined as "OAT" i.e. also oligozoospermic and asthenozoospermic and thus it is possible that the association of teratozoospermia alone was not measured fully. Future studies warrant investigating this further using more chromosome pairs and individuals who display severe teratozoospermia but normal levels of sperm concentration and motility.

## Conclusions

In conclusion we provide evidence that sperm disomy levels can vary considerably between samples from the same man, the reasons for this are unclear but one possible explanation is the involvement of extrinsic factors or lifestyle changes. Such differences provide hope for possible treatment regimes to improve disomy rates. The evidence of correlations between individual semen parameters and increased disomy of individual chromosome pairs, while statistically significant, warrants further investigation. Closer correlations of disomy rates in men with defects in only one of the three criteria used to measure semen quality will form the basis of our future investigations. In future studies it is also likely that we would include a second control group of normal, fertile donors not attending fertility clinics. Ethical considerations precluded this in this case. Through these studies, a closer understanding of the mechanistic basis of the relationship between chromosome segregation and infertility will be achieved.

## Authors' contributions

HGT- performed the majority of FISH experiments, scoring of semen samples, collected data generated within this study and assisted in drafting the manuscript. SH- performed all semen assessments. MD and DC- performed FISH experiments and acted as independent scorers of semen samples. DW- performed statistical analysis. XPZ- provided patient samples with signed informed consent. DKG- conceived the study and participated in its design and drafted the manuscript. All authors have read and approved the final manuscript.
